# Supplementary material for: Cellular transitions during cranial suture establishment in zebrafish
Source: Nat Commun. 2024 Aug 13;15:6948. doi: 10.1038/s41467-024-50780-5 (PMC11322166; doi:10.1038/s41467-024-50780-5)
Supplement: Supplementary file 1 — Supplementary Information [file 41467_2024_50780_MOESM1_ESM.pdf]

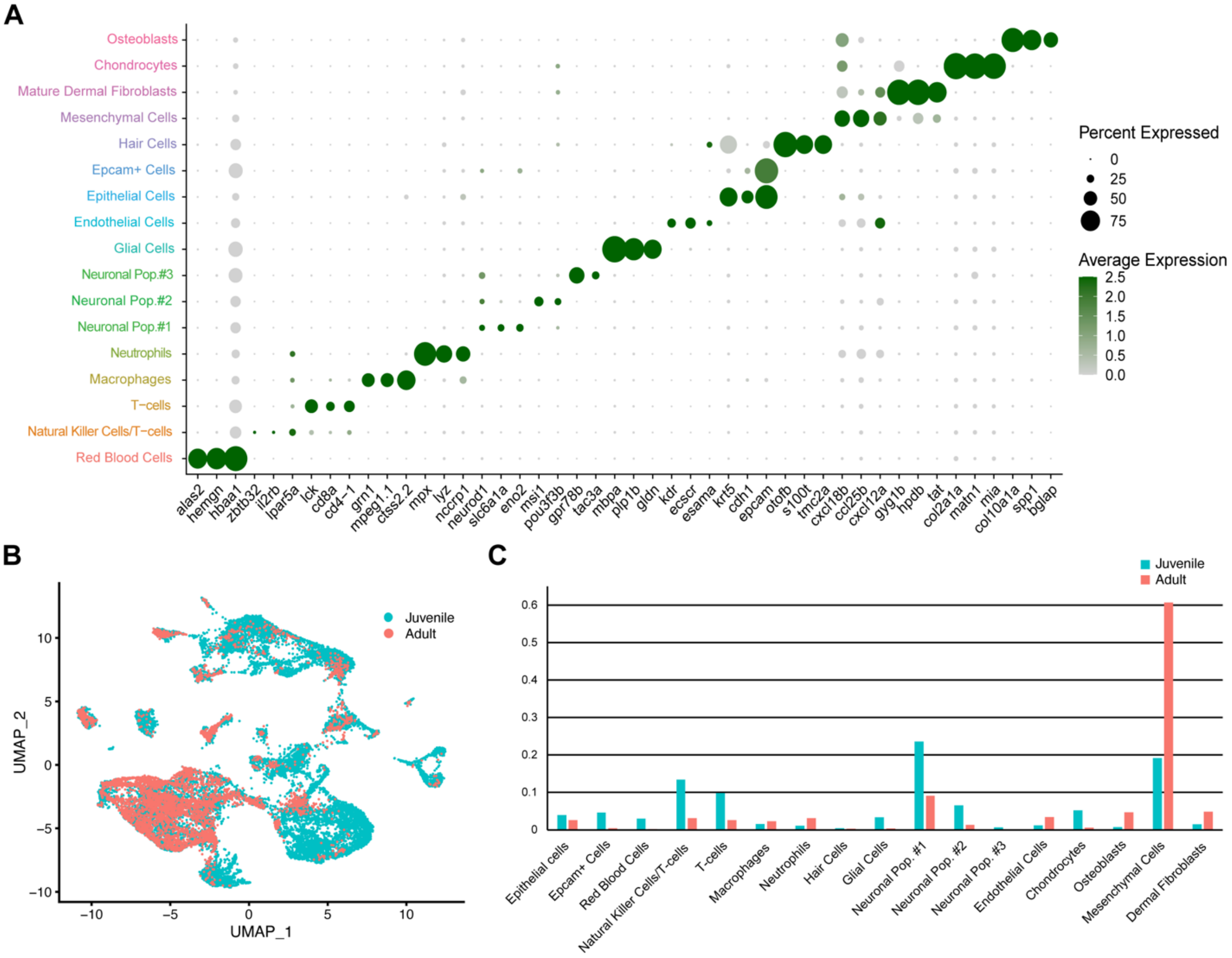

Supplementary Fig. 1. Identification and distribution of cell types in calvaria datasets.

A. Dot plot showing markers enriched for each cluster within the integrated dataset. **B**. Plot of datasets separated by age. **C**. Fractions of total cells from each cluster by age group. Source data are provided as a Source Data file.

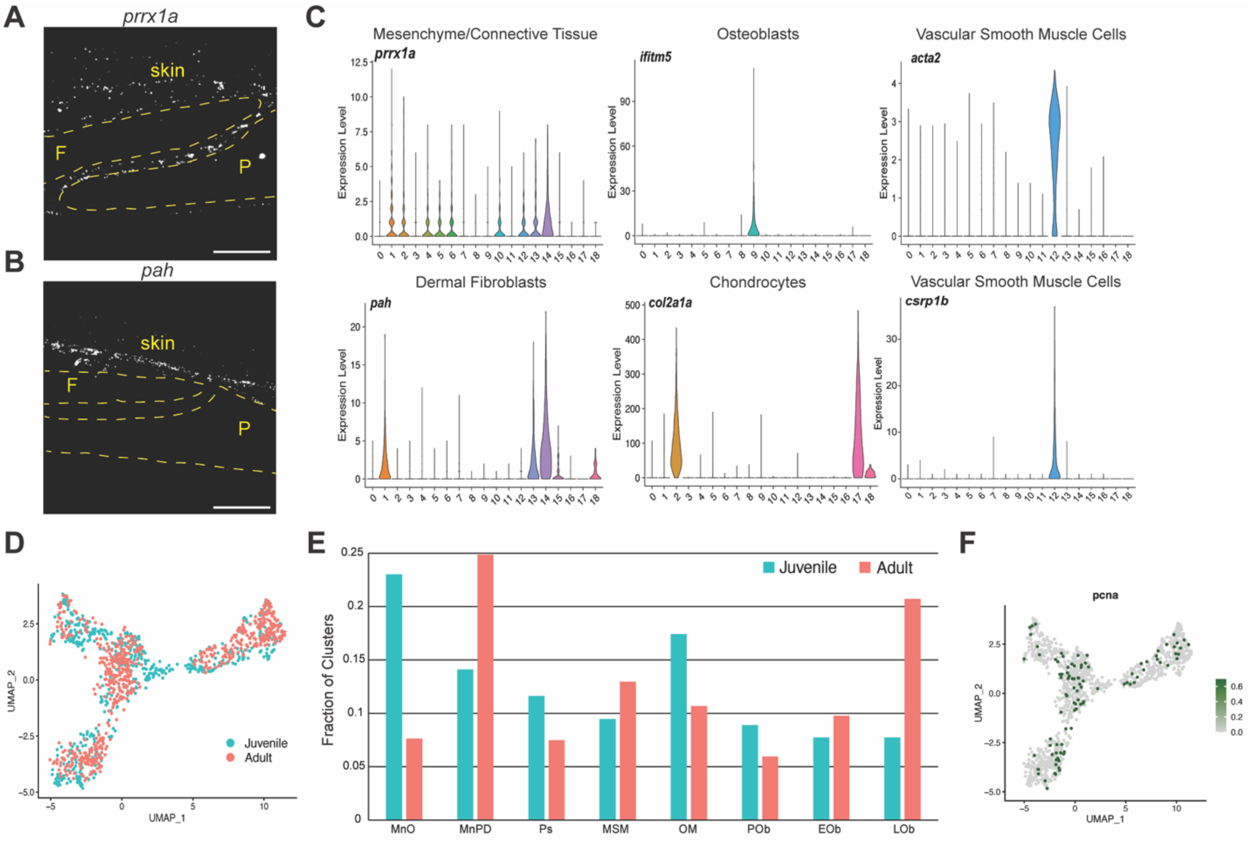

Supplementary Fig. 2. Mesenchyme subsetting strategy and characterization

A, B. In situ mRNA hybridization of adult coronal sutures. Dotted outlines show the frontal (F) and parietal (P) bones. In situs were performed in biological triplicate. Scalebar, 50 μM. **C.** Violin plots of connective and osteogenic markers used to refine the mesenchymal and osteogenic subset. **D**. Plot of *prrx1a*+ mesenchyme and osteoblast subset (Fig. 1E) separated by age. **E**. Fraction of total cells from each cluster by age group. **F**. Plot for *pcna* expression in *prrx1a*+ mesenchyme and osteoblast subset. Source data are provided as a Source Data file.

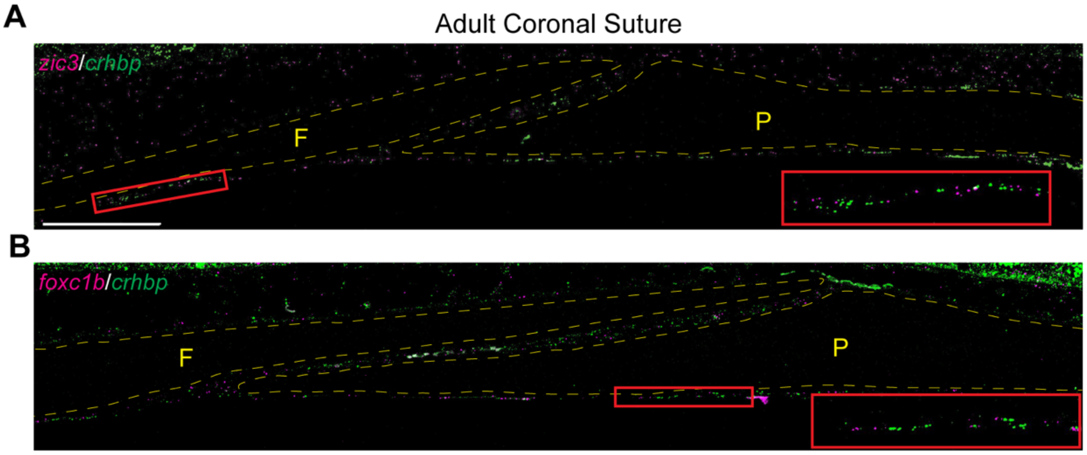

Supplementary Fig. 3. Meningeal marker expression in the adult zebrafish coronal suture.

A,B. Double in situ mRNA hybridization for pan-meninges marker *crhbp* (green) and periosteal dura marker *foxc1b* or meninges-other marker *zic3* (magenta) in the adult coronal suture. Frontal (F) and parietal (P) bones are outlined, and red boxes indicates regions magnified in insets. In situs were performed in biological triplicate. Scalebar: 100 μM.

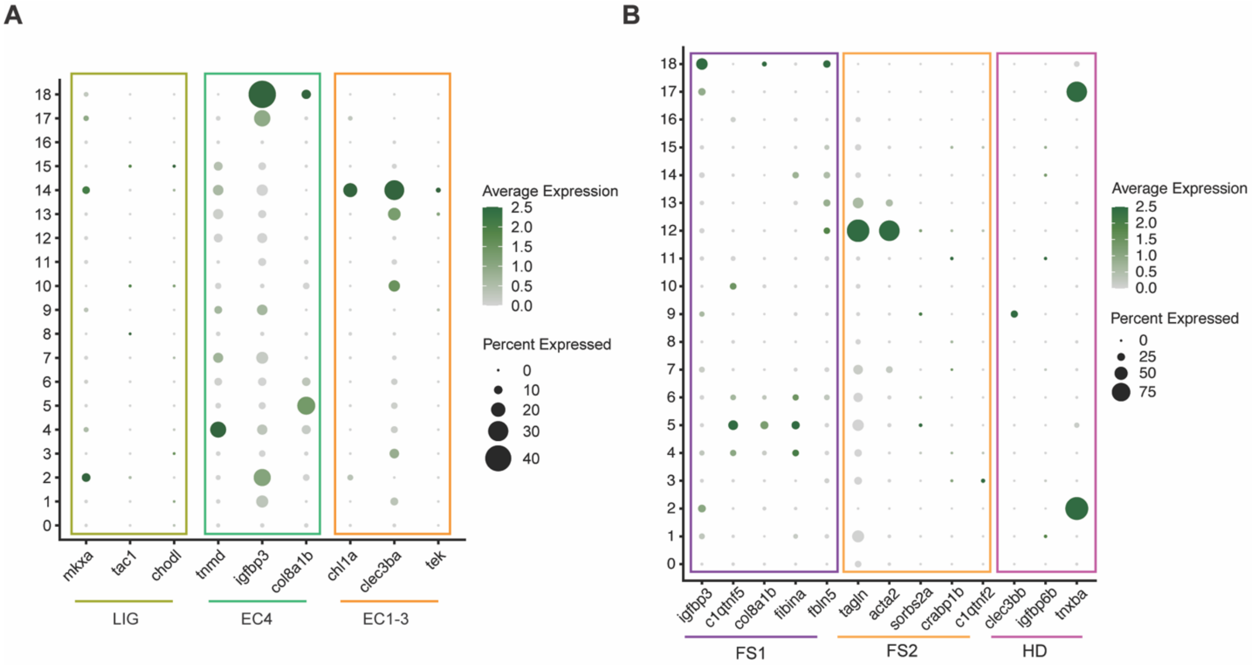

Supplementary Fig. 4. Analysis of ectocranial layers associated with zebrafish calvaria

A. Dot plot showing zebrafish paralogs for markers of ectocranial clusters from the mouse coronal suture dataset mapped onto the connective and skeletogenic subset (Fig. 1C). **B**. Dot plot showing zebrafish paralogs for markers of ectocranial clusters from the mouse frontal suture dataset mapped onto the connective and skeletogenic subset. EC, ectocranial; FS, frontal suture; LIG, ligament-like; HD, hypodermis.

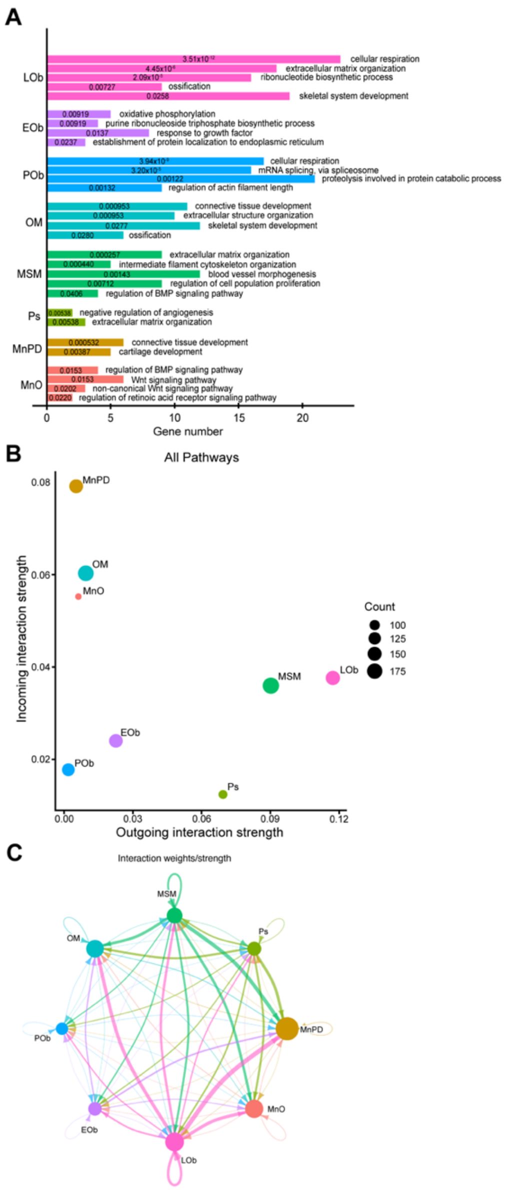

Supplementary Fig. 5. Analysis of sc-RNA-seq captures cluster features and signaling interactions.

A. Plot of representative Gene Ontology (GO) terms enriched within each cluster using significantly enriched genes per cluster. Number of genes present for assigned GO term is on the X-axis and clusters are on the Y-axis. P values are reported within bar graph. **B.** Graphical depiction of the strength of predicted incoming and outcoming signals for each cluster based on CellChat. **C**. Diagram displaying interactions between clusters. Strength of interaction is indicated by the width of the arrow.

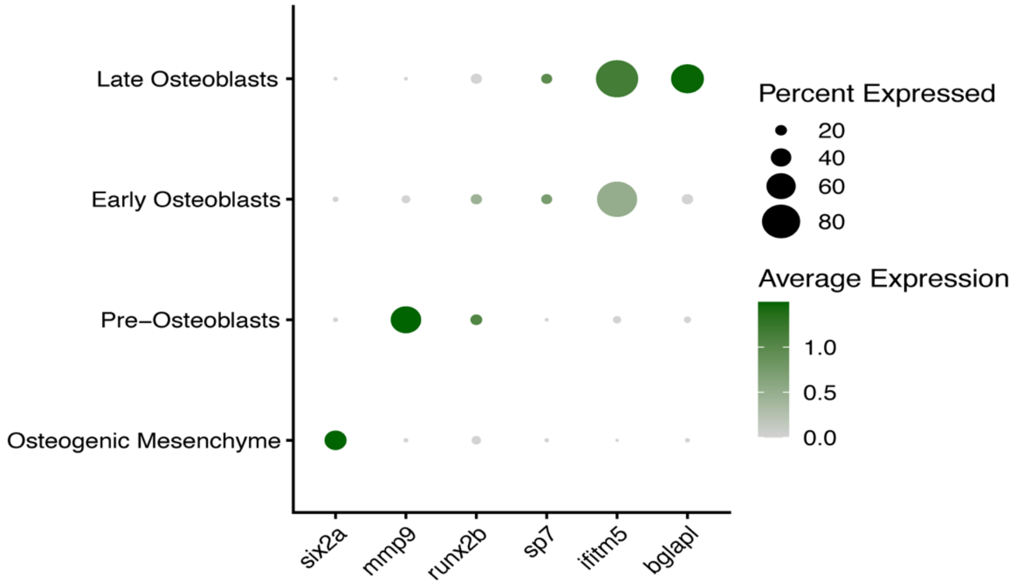

Supplementary Fig. 6. Markers for osteogenic cell types.

Dot plot of markers within the subset used for osteogenic trajectory analysis (see Fig. 3A).

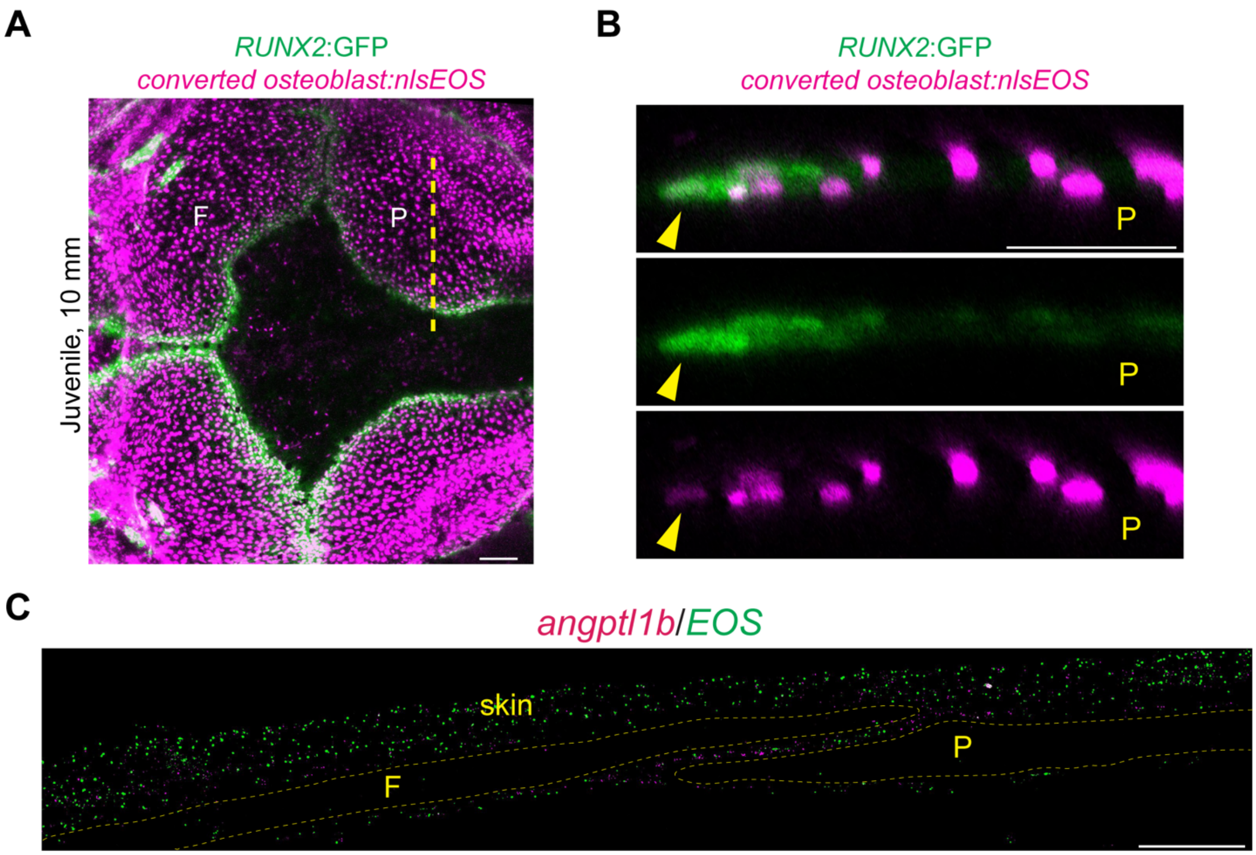

Supplementary Fig. 7. Characterization of *osteoblast*:nlsEOS transgenic line.

A. Confocal image of juvenile skull (dorsal view) shows *osteoblast*:nlsEOS relative to *RUNX2*:GFP+ osteoblasts of the frontal (F) and parietal (P) bones. Heads were subjected to UV-mediated photoconversion of nlsEOS from green to red fluorescence (visualized as magenta). Dotted line marks region for orthogonal section. **B**. Orthogonal section of parietal bone shows overlap of photoconverted *osteoblast*:nlsEOS and *RUNX2*:GFP at the bone front (arrowhead). **C**. Double in situ mRNA hybridization on coronal suture section shows expression of *angptl1b* relative to *nlsEOS*. In situs were performed in biological replicate. Scalebars: 100 μM (A,C), 50 μM (B).

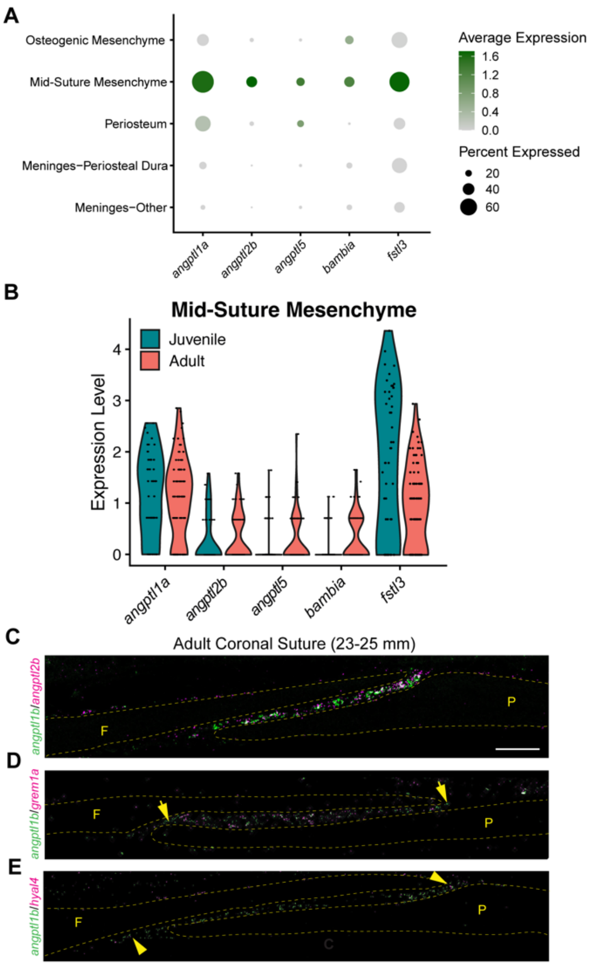

Supplementary Fig. 8. Gene enrichment and marker analysis of regulatory mesenchyme.

A. Dot plot of genes enriched in the regulatory mesenchyme subset. **B.** Violin plot split by age for genes enriched in the regulatory mesenchyme subset. **C-E**. Double in situ analysis of adult coronal suture. Arrowheads mark double-positive cells and arrows mark *angptl1b*+ single cells along the suture. In situs were performed in biological triplicate. Scalebars: 100 μM (A,C).

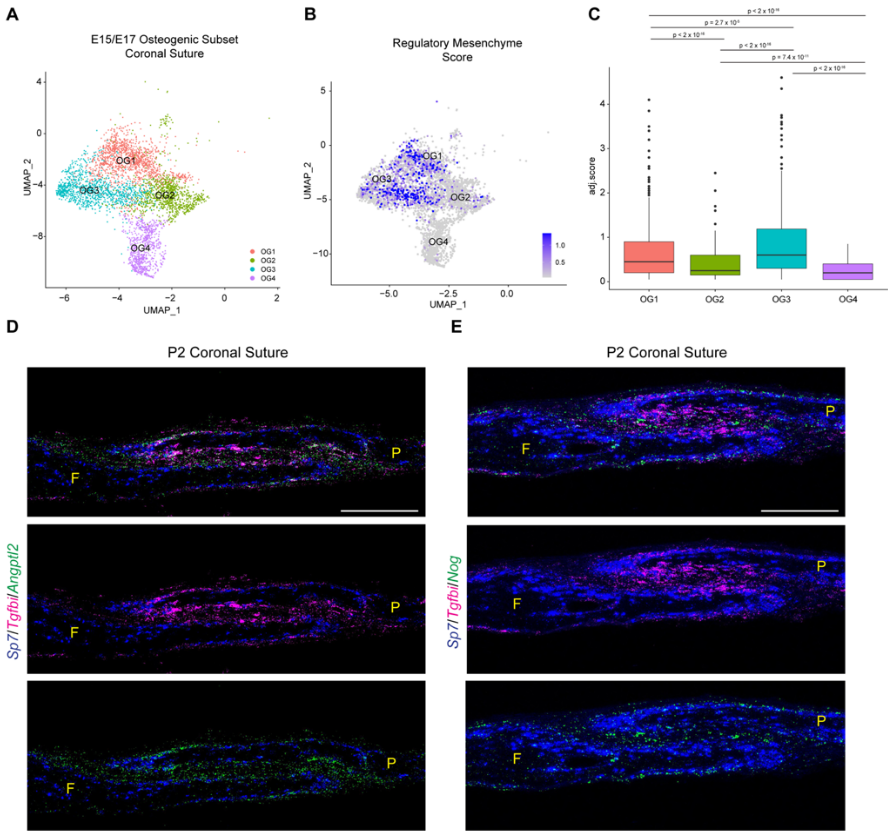

Supplementary Fig. 9. Conserved regulatory mesenchyme program identified in mouse cranial sutures. **A.** UMAP plot of osteogenic subset of E15/E17 coronal suture dataset from [5]. **B**. Module score for regulatory mesenchyme program (*Angptl2*, *Tgfbi*, *Nog*, *Grem1*) plotted on to the osteogenic subset UMAP. **C**. Boxplot of regulatory mesenchyme program module scores for mouse coronal suture osteogenic clusters. The interquartile range (IQR) is defined by the box, the upper and lower borders of each box represent the first and third quartiles, the horizontal line within the box represents the median, and the whiskers represent 1.5*IQR from the first and third quartiles. P value was calculated by a two-sided pairwise Wilcoxon Rank Sum test with Benjamini & Hochberg correction after Kruskal-Wallis test. **D, E.** Triple in situ analysis of postnatal day 2 coronal sutures with frontal (F) and parietal (P) bones indicated. Merged channel is at top with two-color channels below. In situs were performed in biological triplicate. Scalebars: 100 μM (D,E).

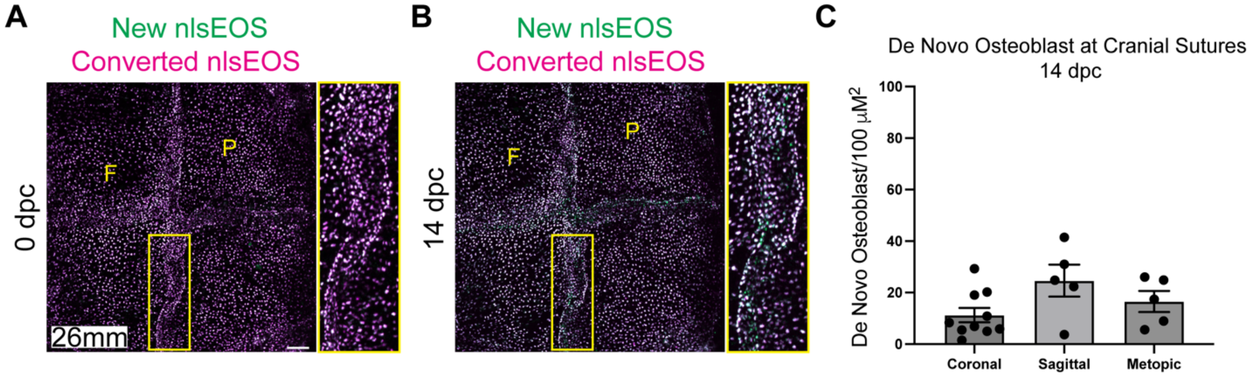

Supplementary Fig. 10. De novo osteoblast differentiation persists in the adult calvaria.

A, B. Serial confocal imaging of individual adult fish (26 mm stage) at 0 (A) and 14 (B) days post-conversion (dpc). Yellow boxes denote magnified regions. **C.** Quantification of newly formed osteoblasts at metopic, coronal, and sagittal sutures. N =10 coronal sutures, N = 5 metopic and sagittal sutures. Scalebars: 100 μM. Error bars represent S.E.M. Source data are provided as a Source Data file.

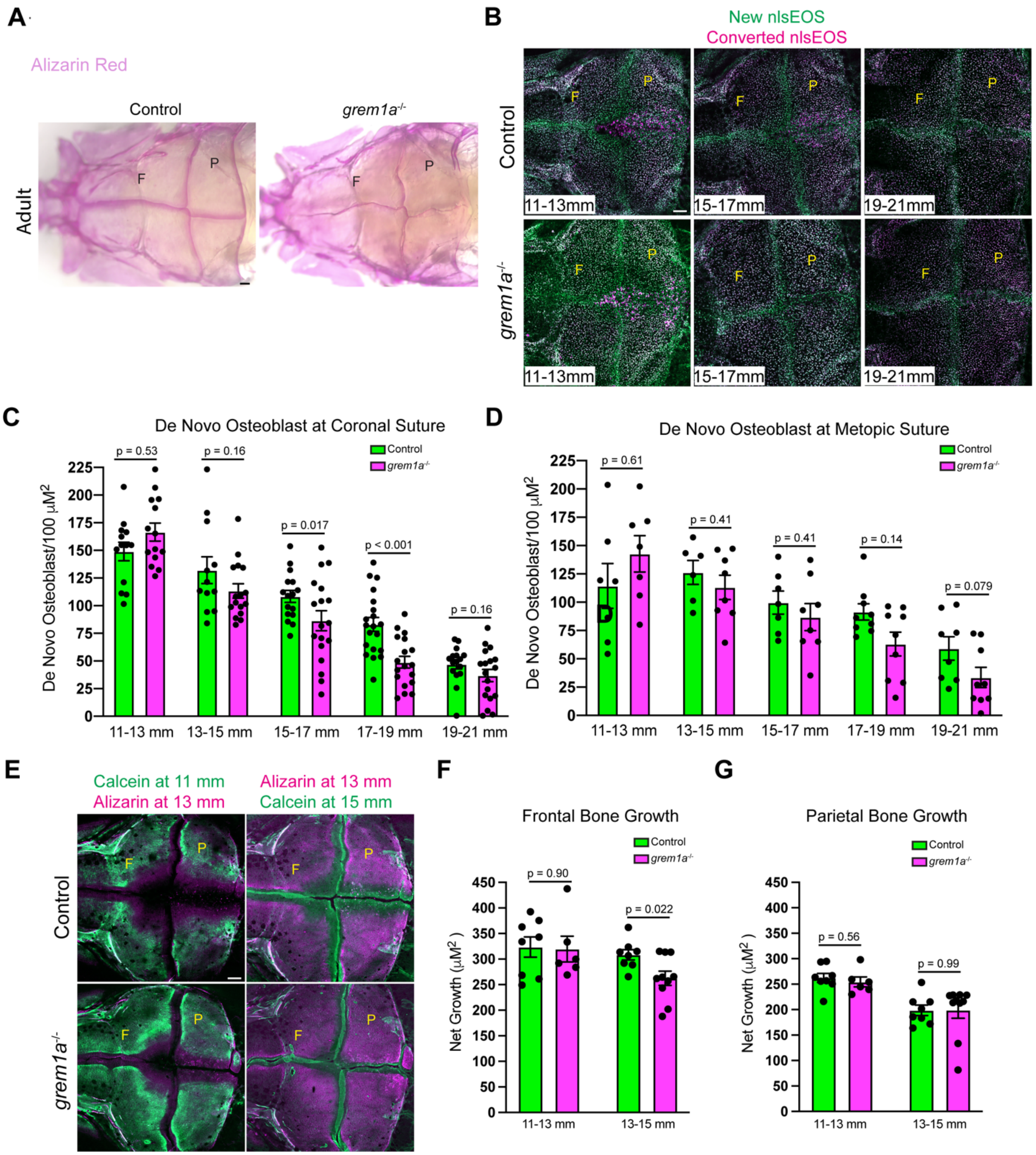

Supplementary Fig. 11. The BMP antagonist grem1a is dispensable for calvaria development.

A. Dissected Alizarin Red stained skullcaps from size matched control and *grem1a*$^{-/-}$ adult fish show established and patent sutures. N = 11 **B.** Serial confocal imaging of individual control and *grem1a*$^{-/-}$ fish in the *osteoblast*:nlsEOS background. Fish were converted beginning at 11 mm, imaged at 13 mm, and then converted again at 13 mm, and the imaging scheme continued until fish reached 21 mm. **C,D**. Quantification of osteoblast addition at metopic and coronal sutures. N = 13 control and N = 14 mutant coronal sutures at 11-13 mm. N = 12 control and N = 16 mutant coronal sutures at 13-15 mm. N = 16 control and N = 18 mutant coronal sutures

at 15-17 mm. N = 20 control and N = 18 mutant coronal sutures at 17-19 mm. N = 16 control and N = 18 mutant coronal sutures at 19-21 mm. N = 7 control and N = 7 mutant metopic sutures at 11-13 mm. N = 6 control and N = 8 mutant metopic sutures at 13-15 mm. N = 7 control and N = 8 mutant metopic sutures at 15-17 mm. N = 9 control and N = 9 mutant metopic sutures at 17-19 mm. N = 8 control and N = 9 mutant metopic sutures at 19-21 mm. **E.** Representative imaging of individual control and *grem1a*$^{-/-}$ zebrafish after repeated staining and imaging using Calcein Green and Alizarin Red stains. **F, G.** Quantification of area of net bone growth between 11-13 mm and 13-15 mm at frontal and parietal bones. N = 8 control frontal bones, N = 6 mutant frontal bones at 11-13 mm. N = 8 control frontal bones, N = 10 mutant frontal bones at 13-15 mm. N = 8 control parietal bones, N = 6 mutant parietal bones at 11-13 mm. N = 8 control parietal bones, N = 10 mutant parietal bones at 13-15 mm. Scalebars: 100 μM. P-values were calculated using a two-tailed non-parametric Student's t-tests. Error bars represent S.E.M. Source data are provided as a Source Data file.
