# Supplementary material for: Cellular transitions during cranial suture establishment in zebrafish
Source: Nat Commun. 2024 Aug 13;15:6948. doi: 10.1038/s41467-024-50780-5 (PMC11322166; doi:10.1038/s41467-024-50780-5)
Supplement: Supplementary file 3 — Description of Additional Supplementary Files [file 41467_2024_50780_MOESM3_ESM.pdf]

# <u>Description of additional supplementary files</u>

**Supplementary Data 1**. Enriched gene list by cluster for whole calvaria dataset (related to Fig. 1B).

**Supplementary Data 2.** Enriched gene list by cluster for connective and skeletogenic subset (related to Fig. 1C).

**Supplementary Data 3.** Enriched gene list by cluster for osteoblast and prrx1a+ mesenchyme subset (related to Fig. 1E).

**Supplementary Data 4.** CellChat Signaling pairs for the prrx1a+ mesenchyme subset.

**Supplementary Data 5.** Genotyping primers for mutant alleles.
