# Supplementary material for: Cellular transitions during cranial suture establishment in zebrafish
Source: Nat Commun. 2024 Aug 13;15:6948. doi: 10.1038/s41467-024-50780-5 (PMC11322166; doi:10.1038/s41467-024-50780-5)
Supplement: Supplementary file 9 — Reporting Summary [file 41467_2024_50780_MOESM9_ESM.pdf]

nature portfolio

Corresponding author(s): D'Juan Farmer
Gage Crump

Last updated by author(s): Jun 11, 2024

Reporting Summary

Nature Portfolio wishes to improve the reproducibility of the work that we publish. This form provides structure for consistency and transparency in reporting. For further information on Nature Portfolio policies, see our Editorial Policies and the Editorial Policy Checklist.

Statistics

For all statistical analyses, confirm that the following items are present in the figure legend, table legend, main text, or Methods section.

n/a	Confirmed	
☐	☒	The exact sample size (*n*) for each experimental group/condition, given as a discrete number and unit of measurement
☐	☒	A statement on whether measurements were taken from distinct samples or whether the same sample was measured repeatedly
☐	☒	The statistical test(s) used AND whether they are one- or two-sided<br>*Only common tests should be described solely by name; describe more complex techniques in the Methods section.*
☒	☐	A description of all covariates tested
☒	☐	A description of any assumptions or corrections, such as tests of normality and adjustment for multiple comparisons
☒	☐	A full description of the statistical parameters including central tendency (e.g. means) or other basic estimates (e.g. regression coefficient) AND variation (e.g. standard deviation) or associated estimates of uncertainty (e.g. confidence intervals)
☒	☐	For null hypothesis testing, the test statistic (e.g. *F*, *t*, *r*) with confidence intervals, effect sizes, degrees of freedom and *P* value noted<br>*Give P values as exact values whenever suitable.*
☒	☐	For Bayesian analysis, information on the choice of priors and Markov chain Monte Carlo settings
☒	☐	For hierarchical and complex designs, identification of the appropriate level for tests and full reporting of outcomes
☒	☐	Estimates of effect sizes (e.g. Cohen's *d*, Pearson's *r*), indicating how they were calculated

Our web collection on statistics for biologists contains articles on many of the points above.

Software and code

Policy information about availability of computer code

Data collection	Juvenile and adult stages were performed in replicates, and sequenced data was aligned using Cellranger v3.0.0 from 10X Genomics against the GRCz11 genome. All parameters were set to their default values. Data analysis was performed with Seurat version 4.1.116.
Data analysis	Datasets were filtered by nFeature_RNA > 200, nFeature_RNA < 2500, and percent.mt < 25. Filtered cells were normalized with SCTransform and datasets were integrated based on the tutorial "Integration and Label Transfer" from Seurat (https://satijalab.org/seurat/archive/v3.0/integration.html). The data were processed using the FindNeighbors() and FindClusters() functions and data were visualized with UMAP (30 principal components). The original dataset was first resolved at a resolution of 0.2 to identify the overall cell types contained within the dataset. The connective and skeletogenic subset was visualized at a resolution of 1.0 and the prrx1a+ mesenchyme and osteoblast subset was visualized at a resolution of 0.8, as these resolution values demonstrated the highest number of clusters with transcriptionally unique signatures. The osteogenic subset of the integrated Seurat object was converted into a Monocle cell dataset and Monocle was performed following the Monocle3 recommended parameters (https://cole-trapnell-lab.github.io/monocle3/docs/trajectories/#learn-graph). The prrx1a + mesenchyme and osteoblast subset was analyzed using the veloctyo51 and CellChat packages24. Scores for regulatory mesenchyme in mouse datasets were assessed and analyzed as previously described5. Mouse datasets were downloaded from Facebase (www.facebase.org, coronal suture, Accession: FB00001236; frontal suture, Accession: FB00001013).

For manuscripts utilizing custom algorithms or software that are central to the research but not yet described in published literature, software must be made available to editors and reviewers. We strongly encourage code deposition in a community repository (e.g. GitHub). See the Nature Portfolio guidelines for submitting code & software for further information.

Data

Policy information about availability of data

All manuscripts must include a data availability statement. This statement should provide the following information, where applicable:

- Accession codes, unique identifiers, or web links for publicly available datasets
- A description of any restrictions on data availability
- For clinical datasets or third party data, please ensure that the statement adheres to our policy

The scRNAseq datasets and the processed Seurat rds objects in this study have been deposited in the Gene Expression Omnibus (GEO) database under accession code GSE223147. Access with private token udcbsoqczbwdfor.

Research involving human participants, their data, or biological material

Policy information about studies with human participants or human data. See also policy information about sex, gender (identity/presentation), and sexual orientation and race, ethnicity and racism.

Reporting on sex and gender	*Use the terms sex (biological attribute) and gender (shaped by social and cultural circumstances) carefully in order to avoid confusing both terms. Indicate if findings apply to only one sex or gender; describe whether sex and gender were considered in study design; whether sex and/or gender was determined based on self-reporting or assigned and methods used. Provide in the source data disaggregated sex and gender data, where this information has been collected, and if consent has been obtained for sharing of individual-level data; provide overall numbers in this Reporting Summary. Please state if this information has not been collected. Report sex- and gender-based analyses where performed, justify reasons for lack of sex- and gender-based analysis.*
Reporting on race, ethnicity, or other socially relevant groupings	*Please specify the socially constructed or socially relevant categorization variable(s) used in your manuscript and explain why they were used. Please note that such variables should not be used as proxies for other socially constructed/relevant variables (for example, race or ethnicity should not be used as a proxy for socioeconomic status). Provide clear definitions of the relevant terms used, how they were provided (by the participants/respondents, the researchers, or third parties), and the method(s) used to classify people into the different categories (e.g. self-report, census or administrative data, social media data, etc.) Please provide details about how you controlled for confounding variables in your analyses.*
Population characteristics	*Describe the covariate-relevant population characteristics of the human research participants (e.g. age, genotypic information, past and current diagnosis and treatment categories). If you filled out the behavioural & social sciences study design questions and have nothing to add here, write "See above."*
Recruitment	*Describe how participants were recruited. Outline any potential self-selection bias or other biases that may be present and how these are likely to impact results.*
Ethics oversight	*Identify the organization(s) that approved the study protocol.*

Note that full information on the approval of the study protocol must also be provided in the manuscript.

Field-specific reporting

Please select the one below that is the best fit for your research. If you are not sure, read the appropriate sections before making your selection.

☒ Life sciences ☐ Behavioural & social sciences ☐ Ecological, evolutionary & environmental sciences

For a reference copy of the document with all sections, see nature.com/documents/nr-reporting-summary-flat.pdf

Life sciences study design

All studies must disclose on these points even when the disclosure is negative.

Sample size	For the RNA analysis experiments at least three zebrafish were evaluated for every probe in order to confirm the consistency of expression patterns. 10-20 animals were used for single cell datasets to achieve sufficient numbers for 10X Genomics.
Data exclusions	Datasets were filtered by nFeature_RNA > 200, nFeature_RNA < 2500, and percent.mt < 25
Replication	Only probes that were reproducible across three embryos were included.
Randomization	No randomization was completed.
Blinding	For quantification of mutants, controls and mutants were coded prior to quantification and decoded after for statistical analysis.

Reporting for specific materials, systems and methods

We require information from authors about some types of materials, experimental systems and methods used in many studies. Here, indicate whether each material, system or method listed is relevant to your study. If you are not sure if a list item applies to your research, read the appropriate section before selecting a response.

Materials & experimental systems

n/a	Involved in the study
☒	☐ Antibodies
☒	☐ Eukaryotic cell lines
☒	☐ Palaeontology and archaeology
☐	☒ Animals and other organisms
☒	☐ Clinical data
☒	☐ Dual use research of concern
☒	☐ Plants

Methods

n/a	Involved in the study
☒	☐ ChIP-seq
☒	☐ Flow cytometry
☒	☐ MRI-based neuroimaging

Animals and other research organisms

Policy information about studies involving animals; ARRIVE guidelines recommended for reporting animal research, and Sex and Gender in Research

Laboratory animals	Published lines include Tg(Mmu.Sox10-Mmu.Fos:Cre)zf384, 43, Tg(actab2:loxP-BFP-STOP-loxP-dsRed)sd27, 44, Tg(fli1a:eGFP)y1, 45, Tg(sp7:mCherry)zf604, 46, Tg(Hsa.RUNX2-Mmu.Fos:EGFP)zf259, 46, TgBAC(mmp9:EGFP)tyt206, 26, cdh1:mlanYFPxt17Tg, 47 , Tg(sp7:EGFP)b1212, 48, tcf12el548, 11, and twist1bel570, 11. Two transgenic lines were created using CRISPR/Cas9-based genomic integration of nlsEOS: grem1a:nlsEOS (guide RNAs targeting the first exon: 5'-AATGGCGCCTTGAAATCCCC-3', 5'-CAGTCACCGCAGACACCGC-3'), angptl1b:nlsEOS (guide RNAs targeting the first intron: 5'-GTCGGTGTGTCGGAGACTGT-3', 5'-TTACACGCTCTCTGCACTCC-3'). Knockout alleles were generated using CRISPR/Cas9 as previously described50 for grem1a (sgRNA, 5'-GGTGCTGGACTCCAGCCAGG-3'), nog2 (sgRNA, 5'-GGAGCACGACCCACGCGAGC-3'), and nog3 (sgRNA, 5'-CGCTCTTTAGGGTCCAGTAC-3'). Genotyping primers are provided in Table S5.
Wild animals	NA
Reporting on sex	When possible, all experiments were included equal numbers of male and female zebrafish. This was not the case for mutants and juvenile stages, when n was limited or gender assessment was impossible.
Field-collected samples	NA
Ethics oversight	All experiments were approved by the Institutional Animal Care and Use Committee at the University of Southern California (Protocol #20771) and the University of California, Los Angeles (ARC-2022-044).

Note that full information on the approval of the study protocol must also be provided in the manuscript.

Plants

Seed stocks	*Report on the source of all seed stocks or other plant material used. If applicable, state the seed stock centre and catalogue number. If plant specimens were collected from the field, describe the collection location, date and sampling procedures.*
Novel plant genotypes	*Describe the methods by which all novel plant genotypes were produced. This includes those generated by transgenic approaches, gene editing, chemical/radiation-based mutagenesis and hybridization. For transgenic lines, describe the transformation method, the number of independent lines analyzed and the generation upon which experiments were performed. For gene-edited lines, describe the editor used, the endogenous sequence targeted for editing, the targeting guide RNA sequence (if applicable) and how the editor was applied.*
Authentication	*Describe any authentication procedures for each seed stock used or novel genotype generated. Describe any experiments used to assess the effect of a mutation and, where applicable, how potential secondary effects (e.g. second site T-DNA insertions, mosiacism, off-target gene editing) were examined.*
